# The South Georgia Medical Association

**Published:** 1875-11

**Authors:** 


THE SOUTH GEORGIA MEDICAL ASSOCIATION met in Bainbridge, Ga., Tuesday, September 21st. It was a fine looking body of men. Dr. Hopkins, of Thomasville, presided. Dr. Thomas, President of the State Board of Health, and Dr. Cromwell, a member of the board for the Second Congressional district, were present.

During the evening session Dr. Cromwell delivered a very able address, and Dr. Thomas, also, made a very happy extemporaneous talk.

Reports were read by Dr. W. N. Bruce, of Bainbridge, on the course, pathology and treatment of "Malarial Hæmaturia;" Dr. T. S. Dekle, of Thomas county, on the "Combined Influence of Sulphate of Quinine and Bromide of Potassium in Malarial

Fever;" and by Dr. Hopkins, a clinical report of six cases of "Uterine Displacement successfully treated by genu-pectoral posture and pneumatic pressure."

